# 1,2-Dimorpholinoethane-1,2-dithione

**DOI:** 10.1107/S1600536809001780

**Published:** 2009-02-11

**Authors:** Yan-Ping Yu, Yuan-Yuan Lin, Bing-Xin Liu

**Affiliations:** a Department of Chemistry, Shanghai University, People’s Republic of China

## Abstract

The title compound, C_10_H_16_N_2_O_2_S_2_, was prepared by a reaction of 4-*tert*-butyl­benzene, morpholine and sulfur. In the crystal structure, both morpholine rings display the typical chair conformation. Weak C—H⋯O hydrogen bonding is present in the crystal structure.

## Related literature

For general background, see: Carmack (1989 ▶). For a related structure, see: Rozentsveig *et al.* (2005 ▶).
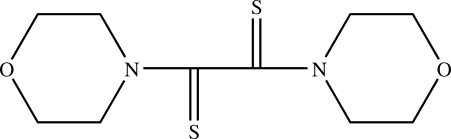


## Experimental

### 

#### Crystal data

C_10_H_16_N_2_O_2_S_2_
*M* *_r_* = 260.37
Monoclinic, 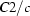

*a* = 34.661 (7) Å
*b* = 6.5155 (12) Å
*c* = 10.6632 (19) Å
β = 93.633 (2)°
*V* = 2403.3 (8) Å^3^
*Z* = 8
Mo *K*α radiation
μ = 0.43 mm^−1^
*T* = 295 (2) K
0.25 × 0.20 × 0.15 mm

#### Data collection

Bruker SMART APEX CCD diffractometer
Absorption correction: multi-scan (*SADABS*; Sheldrick, 1996 ▶) *T*_min_ = 0.905, *T*_max_ = 0.940
6026 measured reflections
2118 independent reflections
1673 reflections with *I* > 2σ(*I*)
*R*_int_ = 0.029

#### Refinement

*R*[*F* ^2^ > 2σ(*F* ^2^)] = 0.035
*wR*(*F* ^2^) = 0.086
*S* = 1.05
2118 reflections
146 parameters
H-atom parameters constrained
Δρ_max_ = 0.19 e Å^−3^
Δρ_min_ = −0.20 e Å^−3^

### 

Data collection: *SMART* (Bruker, 2004 ▶); cell refinement: *SAINT* (Bruker, 2004 ▶); data reduction: *SAINT*; program(s) used to solve structure: *SHELXS97* (Sheldrick, 2008 ▶); program(s) used to refine structure: *SHELXL97* (Sheldrick, 2008 ▶); molecular graphics: *ORTEP-3 for Windows* (Farrugia, 1997 ▶); software used to prepare material for publication: *WinGX* (Farrugia, 1999 ▶).

## Supplementary Material

Crystal structure: contains datablocks I, global. DOI: 10.1107/S1600536809001780/xu2457sup1.cif

Structure factors: contains datablocks I. DOI: 10.1107/S1600536809001780/xu2457Isup2.hkl

Additional supplementary materials:  crystallographic information; 3D view; checkCIF report

## Figures and Tables

**Table 1:** Hydrogen-bond geometry (Å, °)

*D*—H⋯*A*	*D*—H	H⋯*A*	*D*⋯*A*	*D*—H⋯*A*
C2—H2*B*⋯O1^i^	0.97	2.51	3.400 (3)	153

Symmetry code: (i) 
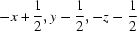
.

**Fig. 1.**
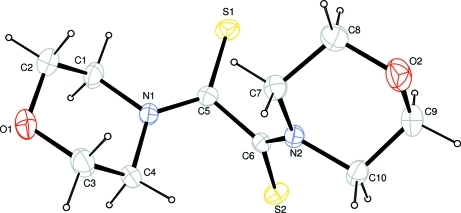
The molecular structure of the title compound with 30% probability displacement ellipsoids.

## supplementary crystallographic information

### Comment

Willgerodt-Kindler reaction is an important synthesize reaction of medicament, but the reaction mechanism is not completely clear (Carmack, 1989). To investigate the reaction mechanism of Willgerodt-Kindler reaction, we performed the reaction of morpholine with 4-tert-butylphenyl and sulfur and obtained single crystals of the title compound. Herein we present its X-ray structure.

The molecular structure of the title compound is shown in Fig. 1. Within the molecule structure, two C═S bond distances are 1.656 (2) Å and 1.666 (2) Å, respectively. The two planes containing the C—S bonds, C1/C4/N1/C5/S1 and C7/C10/N2/C6/S2, are nearly perpendicular to each other with a dihedral angle of 89.94 (7)°. Both morpholino rings display the typical chair conformation, which agrees with that found in the dimorpholine derivative, 4-chloro-N-(2-(4-methylphenyl)-1,2-dimorpholinoethylidene)benzenesulfonamide (Rozentsveig *et al.*,  2005). The adjacent molecules are linked together *via* C—H···O weak hydrogen bonding (Table 1).

### Experimental

The title compound was prepared by a reaction of 4'-*tert*-butylacetophenone (17.72 g, 0.1 mol), morpholine (33 ml, 0.375 mol) and sulfur (5.29 g, 0.165 mol) at 397–405 K until the reaction mixture changed color to puce. Add methanol (100 ml) and active carbon (1 g) into the reaction mixture after the reaction undergoing 10 h. After the reaction mixture cooling to room temperature, the filemot solid product was separated from the reaction mixture. The filemot solid product and was mixed with an ethanol-water solution (1:3) and an aqueous solution (20 ml) of NaOH (0.05 g 1.14 mmol). The mixture was refluxed for 4 h at 357 K and the kelly depositions were obtained from the cooling reaction mixture. The single crystals of the title compound were obtained by recrystallization of the solid product from an ethanol solution after 2 d.

### Refinement

H atoms were placed in calculated positions with C—H = 0.97 Å and included in the final cycles of refinement in riding mode with U_iso_(H) = 1.2U_eq_(C).

### Crystal data

**Table:**

C_10_H_16_N_2_O_2_S_2_	*F*(000) = 1104
*M**_r_* = 260.37	*D*_x_ = 1.439 Mg m^−^^3^
Monoclinic, *C*2/*c*	Mo *K*α radiation, λ = 0.71073 Å
Hall symbol: -C 2yc	Cell parameters from 2010 reflections
*a* = 34.661 (7) Å	θ = 2.0–25.0°
*b* = 6.5155 (12) Å	µ = 0.43 mm^−^^1^
*c* = 10.6632 (19) Å	*T* = 295 K
β = 93.633 (2)°	Prism, colorless
*V* = 2403.3 (8) Å^3^	0.25 × 0.20 × 0.15 mm
*Z* = 8	

### Data collection

**Table:**

Bruker APEX CCD diffractometer	2118 independent reflections
Radiation source: fine-focus sealed tube	1673 reflections with *I* > 2σ(*I*)
graphite	*R*_int_ = 0.029
φ and ω scans	θ_max_ = 25.0°, θ_min_ = 2.4°
Absorption correction: multi-scan (*SADABS*; Sheldrick, 1996)	*h* = −39→40
*T*_min_ = 0.905, *T*_max_ = 0.940	*k* = −7→7
6026 measured reflections	*l* = −12→7

### Refinement

**Table:**

Refinement on *F*^2^	Secondary atom site location: difference Fourier map
Least-squares matrix: full	Hydrogen site location: inferred from neighbouring sites
*R*[*F*^2^ > 2σ(*F*^2^)] = 0.035	H-atom parameters constrained
*wR*(*F*^2^) = 0.086	*w* = 1/[σ^2^(*F*_o_^2^) + (0.0357*P*)^2^ + 1.3632*P*] where *P* = (*F*_o_^2^ + 2*F*_c_^2^)/3
*S* = 1.05	(Δ/σ)_max_ < 0.001
2118 reflections	Δρ_max_ = 0.19 e Å^−^^3^
146 parameters	Δρ_min_ = −0.20 e Å^−^^3^
0 restraints	Extinction correction: *SHELXL97* (Sheldrick, 2008), Fc^*^=kFc[1+0.001xFc^2^λ^3^/sin(2θ)]^-1/4^
Primary atom site location: structure-invariant direct methods	Extinction coefficient: 0.0028 (3)

### Special details

**Table:**

Geometry. All e.s.d.'s (except the e.s.d. in the dihedral angle between two l.s. planes) are estimated using the full covariance matrix. The cell e.s.d.'s are taken into account individually in the estimation of e.s.d.'s in distances, angles and torsion angles; correlations between e.s.d.'s in cell parameters are only used when they are defined by crystal symmetry. An approximate (isotropic) treatment of cell e.s.d.'s is used for estimating e.s.d.'s involving l.s. planes.
Refinement. Refinement of *F*^2^ against ALL reflections. The weighted *R*-factor *wR* and goodness of fit *S* are based on *F*^2^, conventional *R*-factors *R* are based on *F*, with *F* set to zero for negative *F*^2^. The threshold expression of *F*^2^ > σ(*F*^2^) is used only for calculating *R*-factors(gt) *etc*. and is not relevant to the choice of reflections for refinement. *R*-factors based on *F*^2^ are statistically about twice as large as those based on *F*, and *R*- factors based on ALL data will be even larger.

### Fractional atomic coordinates and isotropic or equivalent isotropic displacement parameters (Å^2^)

**Table:**

	*x*	*y*	*z*	*U*_iso_*/*U*_eq_	
S1	0.372884 (17)	−0.19547 (8)	0.11699 (5)	0.0400 (2)	
S2	0.380220 (17)	0.28649 (9)	0.28638 (5)	0.04075 (19)	
N1	0.32643 (5)	0.1044 (3)	0.03467 (16)	0.0338 (4)	
N2	0.41166 (5)	0.2706 (3)	0.06457 (16)	0.0322 (4)	
O1	0.27344 (4)	0.2067 (3)	−0.16300 (16)	0.0515 (5)	
O2	0.48123 (4)	0.2411 (3)	−0.04519 (17)	0.0557 (5)	
C1	0.29717 (6)	−0.0402 (4)	−0.0084 (2)	0.0458 (6)	
H1A	0.2748	−0.0269	0.0412	0.055*	
H1B	0.3070	−0.1790	0.0014	0.055*	
C2	0.28575 (7)	0.0009 (4)	−0.1446 (2)	0.0504 (6)	
H2A	0.3076	−0.0258	−0.1947	0.060*	
H2B	0.2650	−0.0913	−0.1727	0.060*	
C3	0.30297 (7)	0.3439 (4)	−0.1261 (2)	0.0489 (6)	
H3A	0.2940	0.4833	−0.1412	0.059*	
H3B	0.3249	0.3208	−0.1765	0.059*	
C4	0.31559 (6)	0.3191 (3)	0.0111 (2)	0.0400 (6)	
H4A	0.3375	0.4079	0.0327	0.048*	
H4B	0.2947	0.3573	0.0626	0.048*	
C5	0.35897 (6)	0.0447 (3)	0.09058 (18)	0.0294 (5)	
C6	0.38568 (6)	0.2090 (3)	0.13964 (18)	0.0288 (5)	
C7	0.41350 (6)	0.2047 (3)	−0.0666 (2)	0.0356 (5)	
H7A	0.4101	0.3225	−0.1218	0.043*	
H7B	0.3928	0.1081	−0.0878	0.043*	
C8	0.45103 (6)	0.1074 (4)	−0.0844 (2)	0.0490 (6)	
H8A	0.4532	−0.0187	−0.0361	0.059*	
H8B	0.4529	0.0733	−0.1724	0.059*	
C9	0.47931 (6)	0.2926 (4)	0.0832 (2)	0.0508 (6)	
H9A	0.5013	0.3783	0.1093	0.061*	
H9B	0.4809	0.1682	0.1333	0.061*	
C10	0.44318 (6)	0.4028 (4)	0.1069 (2)	0.0433 (6)	
H10A	0.4421	0.4320	0.1958	0.052*	
H10B	0.4418	0.5315	0.0611	0.052*	

### Atomic displacement parameters (Å^2^)

**Table:**

	*U*^11^	*U*^22^	*U*^33^	*U*^12^	*U*^13^	*U*^23^
S1	0.0510 (4)	0.0286 (3)	0.0395 (4)	0.0032 (3)	−0.0054 (3)	0.0015 (2)
S2	0.0501 (4)	0.0399 (3)	0.0323 (3)	−0.0051 (3)	0.0034 (3)	−0.0076 (2)
N1	0.0288 (9)	0.0299 (9)	0.0418 (11)	−0.0012 (8)	−0.0053 (8)	0.0005 (8)
N2	0.0271 (9)	0.0358 (10)	0.0336 (10)	−0.0022 (8)	0.0007 (7)	−0.0031 (8)
O1	0.0382 (9)	0.0582 (11)	0.0560 (11)	0.0050 (8)	−0.0147 (7)	0.0016 (8)
O2	0.0339 (9)	0.0722 (12)	0.0620 (12)	−0.0023 (8)	0.0117 (8)	−0.0043 (9)
C1	0.0345 (12)	0.0426 (14)	0.0587 (16)	−0.0099 (11)	−0.0092 (11)	0.0003 (11)
C2	0.0424 (14)	0.0558 (16)	0.0512 (16)	−0.0012 (12)	−0.0115 (11)	−0.0101 (12)
C3	0.0402 (13)	0.0461 (15)	0.0594 (16)	0.0046 (12)	−0.0054 (11)	0.0089 (12)
C4	0.0301 (11)	0.0351 (12)	0.0539 (15)	0.0058 (10)	−0.0048 (10)	−0.0021 (10)
C5	0.0327 (11)	0.0323 (11)	0.0234 (11)	0.0004 (9)	0.0030 (8)	−0.0003 (9)
C6	0.0273 (10)	0.0266 (11)	0.0320 (12)	0.0036 (9)	−0.0032 (9)	0.0025 (9)
C7	0.0347 (12)	0.0405 (13)	0.0316 (12)	0.0002 (10)	0.0013 (9)	0.0012 (10)
C8	0.0437 (14)	0.0554 (16)	0.0486 (15)	0.0028 (12)	0.0090 (11)	−0.0080 (12)
C9	0.0314 (13)	0.0636 (17)	0.0571 (17)	−0.0067 (12)	−0.0003 (11)	0.0028 (13)
C10	0.0344 (12)	0.0436 (14)	0.0517 (15)	−0.0106 (11)	0.0020 (10)	−0.0075 (11)

### Geometric parameters (Å, °)

**Table:**

S1—C5	1.656 (2)	C3—C4	1.509 (3)
S2—C6	1.666 (2)	C3—H3A	0.9700
N1—C5	1.301 (2)	C3—H3B	0.9700
N1—C1	1.438 (3)	C4—H4A	0.9700
N1—C4	1.466 (3)	C4—H4B	0.9700
N2—C6	1.305 (3)	C5—C6	1.488 (3)
N2—C10	1.441 (2)	C7—C8	1.470 (3)
N2—C7	1.468 (3)	C7—H7A	0.9700
O1—C3	1.397 (3)	C7—H7B	0.9700
O1—C2	1.417 (3)	C8—H8A	0.9700
O2—C8	1.405 (3)	C8—H8B	0.9700
O2—C9	1.415 (3)	C9—C10	1.479 (3)
C1—C2	1.505 (3)	C9—H9A	0.9700
C1—H1A	0.9700	C9—H9B	0.9700
C1—H1B	0.9700	C10—H10A	0.9700
C2—H2A	0.9700	C10—H10B	0.9700
C2—H2B	0.9700		
			
C5—N1—C1	121.59 (18)	H4A—C4—H4B	108.3
C5—N1—C4	124.67 (17)	N1—C5—C6	116.62 (18)
C1—N1—C4	113.73 (16)	N1—C5—S1	126.50 (16)
C6—N2—C10	122.03 (18)	C6—C5—S1	116.84 (14)
C6—N2—C7	124.58 (17)	N2—C6—C5	116.33 (18)
C10—N2—C7	113.29 (17)	N2—C6—S2	127.33 (16)
C3—O1—C2	110.98 (16)	C5—C6—S2	116.28 (15)
C8—O2—C9	110.79 (18)	N2—C7—C8	109.97 (17)
N1—C1—C2	109.19 (19)	N2—C7—H7A	109.7
N1—C1—H1A	109.8	C8—C7—H7A	109.7
C2—C1—H1A	109.8	N2—C7—H7B	109.7
N1—C1—H1B	109.8	C8—C7—H7B	109.7
C2—C1—H1B	109.8	H7A—C7—H7B	108.2
H1A—C1—H1B	108.3	O2—C8—C7	110.04 (19)
O1—C2—C1	111.12 (19)	O2—C8—H8A	109.7
O1—C2—H2A	109.4	C7—C8—H8A	109.7
C1—C2—H2A	109.4	O2—C8—H8B	109.7
O1—C2—H2B	109.4	C7—C8—H8B	109.7
C1—C2—H2B	109.4	H8A—C8—H8B	108.2
H2A—C2—H2B	108.0	O2—C9—C10	111.87 (19)
O1—C3—C4	111.5 (2)	O2—C9—H9A	109.2
O1—C3—H3A	109.3	C10—C9—H9A	109.2
C4—C3—H3A	109.3	O2—C9—H9B	109.2
O1—C3—H3B	109.3	C10—C9—H9B	109.2
C4—C3—H3B	109.3	H9A—C9—H9B	107.9
H3A—C3—H3B	108.0	N2—C10—C9	106.86 (19)
N1—C4—C3	108.89 (18)	N2—C10—H10A	110.4
N1—C4—H4A	109.9	C9—C10—H10A	110.4
C3—C4—H4A	109.9	N2—C10—H10B	110.4
N1—C4—H4B	109.9	C9—C10—H10B	110.4
C3—C4—H4B	109.9	H10A—C10—H10B	108.6

### Hydrogen-bond geometry (Å, °)

**Table:**

*D*—H···*A*	*D*—H	H···*A*	*D*···*A*	*D*—H···*A*
C2—H2B···O1^i^	0.97	2.51	3.400 (3)	153

Symmetry codes: (i) −*x*+1/2, *y*−1/2, −*z*−1/2.
